# Genome-Wide Analysis of *Haemonchus contortus* Proteases and Protease Inhibitors Using Advanced Informatics Provides Insights into Parasite Biology and Host–Parasite Interactions

**DOI:** 10.3390/ijms241512320

**Published:** 2023-08-01

**Authors:** Yuanting Zheng, Neil D. Young, Jiangning Song, Robin B. Gasser

**Affiliations:** 1 Melbourne Veterinary School, Faculty of Science, The University of Melbourne, Parkville, VIC 3010, Australia; yuantingz@student.unimelb.edu.au; 2 Department of Data Science and AI, Faculty of IT, Monash University, Melbourne, VIC 3800, Australia; jiangning.song@monash.edu; 3 Department of Biochemistry and Molecular Biology, Biomedicine Discovery Institute, Monash University, Melbourne, VIC 3800, Australia; 4 Monash Data Futures Institute, Monash University, Melbourne, VIC 3800, Australia

**Keywords:** parasitic nematode, *Haemonchus contortus*, proteome, protease, protease inhibitor, genome-wide identification, classification and annotation, bioinformatic workflow

## Abstract

Biodiversity within the animal kingdom is associated with extensive molecular diversity. The expansion of genomic, transcriptomic and proteomic data sets for invertebrate groups and species with unique biological traits necessitates reliable in silico tools for the accurate identification and annotation of molecules and molecular groups. However, conventional tools are inadequate for lesser-known organismal groups, such as eukaryotic pathogens (parasites), so that improved approaches are urgently needed. Here, we established a combined sequence- and structure-based workflow system to harness well-curated publicly available data sets and resources to identify, classify and annotate proteases and protease inhibitors of a highly pathogenic parasitic roundworm (nematode) of global relevance, called *Haemonchus contortus* (barber’s pole worm). This workflow performed markedly better than conventional, sequence-based classification and annotation alone and allowed the first genome-wide characterisation of protease and protease inhibitor genes and gene products in this worm. In total, we identified 790 genes encoding 860 proteases and protease inhibitors representing 83 gene families. The proteins inferred included 280 metallo-, 145 cysteine, 142 serine, 121 aspartic and 81 “mixed” proteases as well as 91 protease inhibitors, all of which had marked physicochemical diversity and inferred involvements in >400 biological processes or pathways. A detailed investigation revealed a remarkable expansion of some protease or inhibitor gene families, which are likely linked to parasitism (e.g., host–parasite interactions, immunomodulation and blood-feeding) and exhibit stage- or sex-specific transcription profiles. This investigation provides a solid foundation for detailed explorations of the structures and functions of proteases and protease inhibitors of *H. contortus* and related nematodes, and it could assist in the discovery of new drug or vaccine targets against infections or diseases.

## 1. Introduction

Proteases are enzymes that play vital roles in breaking down proteins into peptides or amino acids through the cleavage of peptide bonds and/or the catalysation of hydrolysis reactions [1]. They are actively involved in many crucial biological processes in organisms, such as immune responses [2,3], the digestion of food proteins [4] and programmed cell death (apoptosis) [5,6]. Moreover, the molecular and functional variations in proteases (and their inhibitors) in different species are inextricably linked to the evolution and biodiversity of organisms [1,7].

Proteases can be divided into the following main classes: asparagine, aspartic, cysteine, glutamic, metallo-, serine, threonine and ‘mixed’ proteases [8]. The online MEROPS database (https://www.ebi.ac.uk/merops/index.shtml; accessed on 15 February 2023); ref. [9]) is a comprehensive repository of information on proteases, such as classifications (classes), identifiers, structures, cleavage sites and protease inhibitors, enabling the study of many aspects of proteases [8]. Investigations of proteases of eukaryotic parasites are particularly important because of the critical roles that these enzymes play in pathogen invasion and tissue penetration [10], parasite growth, development and reproduction [11], as well as the acquisition/digestion of nutrients by parasites [12] and the modulation of immune responses and disease by parasites in host animals [13,14].

Research on parasite-derived proteases has been an active area for more than 70 years (reviewed by [15,16,17,18,19,20,21]). Recent advances in this field have expanded our understanding of the diversity and specificity of parasite-derived proteases, with the identification of unique proteases in various species of parasitic helminths, such as *Angiostrongylus cantonensis* [22], *Ascaris suum* [23] and *Meloidogyne incognita* [24]. These studies have highlighted the potential of proteases as potential drug and vaccine targets for the development of novel interventions against parasites (e.g., [18,21]). The use of advanced molecular technologies and informatics [25,26] is now providing improved insights into parasite-derived proteases, such as their classification and involvement in biological processes, as well as host–parasite interactions, with potential implications for the design of protease inhibitors.

Some of our research focuses on parasitic helminths with significant veterinary economic impact, particularly nematodes such as *Haemonchus contortus* (e.g., [27,28,29,30,31,32]). This parasite infects particularly small ruminants worldwide, and it causes substantial disease (haemonchosis) and production losses to the farming and animal industries [33,34,35]. Current control of haemonchosis is heavily reliant on anthelmintics, and the available vaccine does not protect animals for extended periods [36]. In addition, drug resistance in *H. contortus* poses a significant threat and demands the discovery of new interventions [37,38], which might be guided by molecular investigations.

There have been major advances in the genomics, transcriptomics and proteomics of *H. contortus* [39,40,41,42,43,44,45,46,47,48], allowing detailed explorations of this parasite at the molecular level and revealing distinct biological mechanisms and/or critical pathways [32,49]. Some recently developed informatic pipelines have demonstrated utility for analyses of molecular data sets [25,50] as well as protein annotation [51] and the prediction of essential genes [52]. In this context, exploring the proteases and associated inhibitors in *H. contortus* using abundant omics data sets and advanced informatics could contribute to finding parasite-specific targets.

In contrast to model organisms (e.g., *Caenorhabditis elegans* and *Drosophila melanogaster*), which possess a vast array of proteases [53,54,55], there has been no profound, systematic classification of proteases of *H. contortus* at the whole-genome level, despite previous studies of selected groups of proteases using traditional molecular techniques (e.g., [56,57,58,59,60]). There has been limited information on the transcription/expression of protease genes in *H. contortus* across different developmental stages and both sexes as well as the structures of proteases and their inhibitors, and conventional informatic methods [61] have not been sufficiently reliable for genome-wide identification and annotation of proteases and their inhibitors. In this study, we aimed to systematically identify, classify and annotate proteases and protease inhibitors encoded in the *H. contortus* genome using an advanced informatic workflow (employing a sequence- and structure-based strategy), paving the way for a better understanding of these molecules and providing potential for the discovery of new intervention (drug or vaccine) targets.

## 2. Results

### 2.1. Proteases and Protease Inhibitors Encoded in the H. contortus Genome

**Figure 1:**
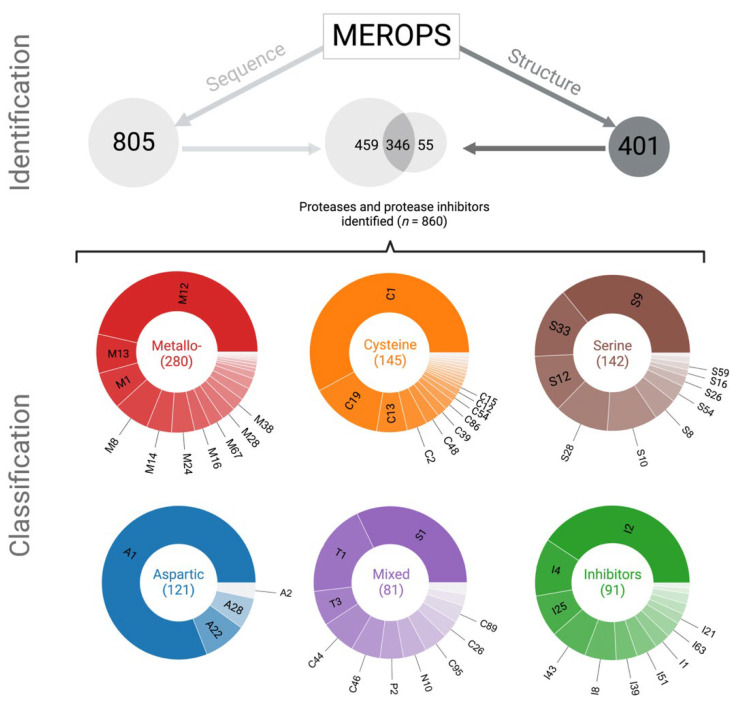
Identification and classification of proteases and protease inhibitors encoded in the *Haemonchus contortus* genome employing a combined sequence- and structure-based approach. Using curated sequence data from the MEROPS database, we identified 805 encoded proteins based on sequence homology (left). Based on structural information available in the MEROPS database, we downloaded protein structures of proteases and protease inhibitors from the PDB database and used US-align to identify 401 proteins (right). Protein structures were predicted using the program AlphaFold2; only structures with a pLDDT value of >70 were retained. By merging the results obtained via sequence and structure searches, we identified a total of 860 proteins, 346 of which were identified based on both sequence and structure. These 860 proteins represented five distinct classes of proteases (pie charts: 280 metallo-, 145 cysteine, 142 serine, 121 aspartic and 81 ‘mixed’ proteases) as well as 91 protease inhibitors (green). The pie charts in the figure show the different gene families within individual classes; the top 10 gene families are designated in black.

Using a combined sequence- and structure-based approach (Figure 1), we identified 790 genes in the *H. contortus* genome encoding 860 proteases and protease inhibitors (including isoforms), which were assigned to 83 distinct families (Table 1). These molecules included 280 metallo-, 145 cysteine, 142 serine, 121 aspartic and 81 ‘mixed’ proteases as well as 91 protease inhibitors (Figure 1). Of these 860 proteins, 346 (40.2%) were identified using both sequence-based and structure-based methods, whereas 55 (6.4%) proteins were identified using a structure-based and 459 (53.4%) employing a sequence-based method alone (Figure 1). Of all 860 protease and protease inhibitors inferred, 256 proteins had been identified in previous LC–MS/MS-based studies of the somatic and excretory/secretory proteomes of *H. contortus* [43,44], and 53 that were phosphorylated (Table S1) might play a role in the regulation of protease activity.

The density of genes encoding proteases or protease inhibitors was highest on chromosome chr1 (*n* = 196) and lowest on the sex chromosome chrX (*n* = 55) (Figure S1). Metallo-protease genes (*n* > 50) were shown to be abundant on individual chromosomes, except chr1, in which cysteine protease genes predominated (*n* = 77) (Figure S1).

### 2.2. Proteases and Protease Inhibitors Represent at Least 83 Gene Families 

In *H. contortus*, proteases are represented by a diverse range of gene families. Metallo-proteases (*n* = 280) are encoded by 21 gene families. The top three gene families account for more than half of all metallo-proteases, with the M12 family being the largest (130; 46.4%), followed by families M13 and M1. In contrast, aspartic proteases are represented by four gene families, with family A1 comprising 81% (98) of them (Figure 1). Cysteine proteases (*n* = 145) were encoded by 20 gene families; more than half of these proteases belong to family C1, and the remainder are in 11 other families—with one protease per family. Serine proteases were relatively evenly distributed among the 12 gene families, except for family S9 with most serine proteases (*n* = 51; 35.9%) (Figure 1). Serine and mixed proteases both represent 12 gene families; most of the latter proteases (*n* = 81) belong to families S1 and T1. Protease inhibitors represent 14 gene families, with I2, I4 and I25 containing most protease inhibitors (i.e., 40.7%, 12.1% and 8.8%, respectively) (Figure 1).

### 2.3. Protease Groups Have Divergent Biophysical Properties

Individual biophysical properties predicted for *H. contortus* proteases vary significantly, depending on their classification. Aspartic proteases had the lowest average molecular weight (46.75 kDa) and pI (5.78), whereas metallo-proteases had the highest pI (7.11) and the lowest mean charge (−1.63) at pH 7.0. Cysteine proteases had the lowest average GRAVY (−0.40) and the broadest range of charge values (−79.06 to 36.3) at pH 7.0. Serine proteases had an average pI (6.89) and mean GRAVY (−0.22), and mixed proteases had a slightly lower mean GRAVY (−0.25) than cysteine proteases. Moreover, protease inhibitors shared similar molecular weights and pIs to proteases, but exhibited a broader range of charges (−96.51 to 29.66) at pH 7.0 (Table S1).

### 2.4. Marked Variability in the Presence/Absence of a Signal Peptide

**Figure 2:**
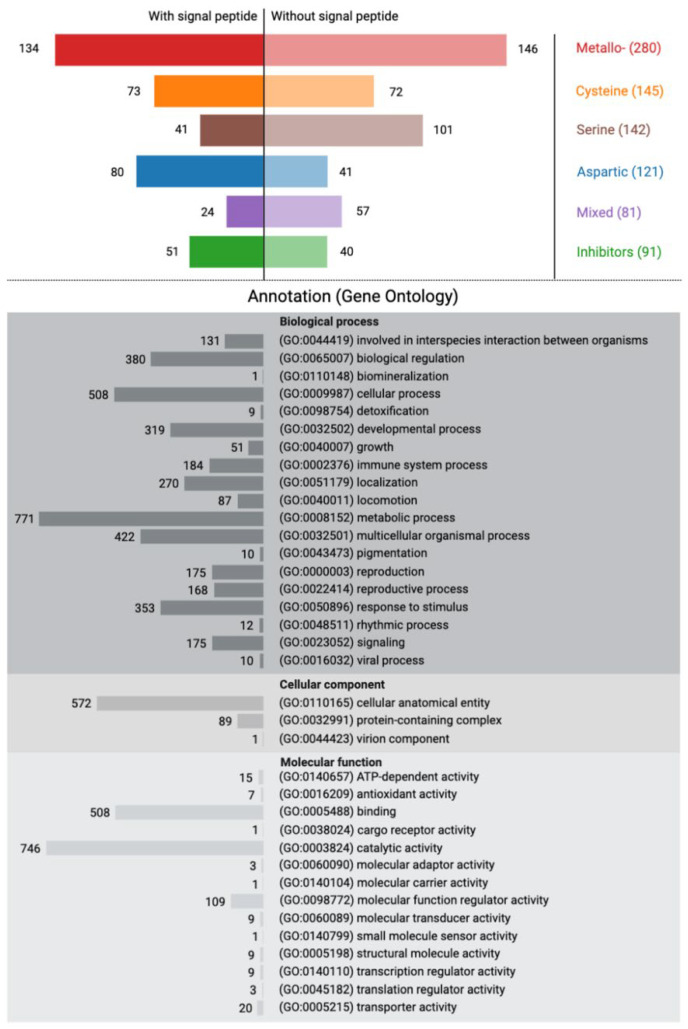
*Haemonchus contortus* proteases and protease inhibitors, and their annotations. At the top, the numbers of proteins with or without signal peptides are given as well as total numbers (right). At the bottom, all proteins were assigned the GO annotations, according to biological process (BP), cellular component (CC) and/or molecular function (MF) (cf. Table S2).

The number of proteins with signal peptides varied among the different groups of proteases. Almost half of all metallo-proteases (i.e., 134 of 280) had signal peptides, followed by 66.1% aspartic proteases (80 of 121). Cysteine and serine proteases had fewer proteins with signal peptides—i.e., 50.3% (73 of 145) and 28.9% (41 of 142), respectively. The mixed proteases had the least proteins with signal peptides (24 of 81) (Figure 2). These findings suggest that the presence/absence of signal peptides could relate to different biological roles and/or transport of distinct protein groups, and signal peptides were more prevalent in some groups than others (e.g., 66.1% for aspartic vs. 28.9% for serine proteases). The percentage of protein inhibitors with signal peptides (56.0%; 51 of 91) was similar to that of cysteine proteases (50.3%; 73 of 145) (Figure 2).

### 2.5. GO Annotations and Key Pathways 

In total, 790 *H. contortus* genes encoding proteases or protease inhibitors were assigned 5560 GO terms (level 2) linked to 19 biological processes (BPs), three cellular components (CCs) and 14 molecular functions (MFs) (Table S2). Metabolic processes (GO:0008152) and cellular anatomical entity (GO:0110165) were the largest sub-categories in BP and CC, respectively. For MF, catalytic activity (GO:0003824) and binding (GO:0005488) were the predominant annotated functions (Figure 2).

Of the 790 *H. contortus* genes encoding proteases and protease inhibitors, 340 have *C. elegans* orthologues encoding 11 (9.6%) aspartic, 48 (36.1%) serine, 111 (42.9%) metallo-, 58 (80.6%) mixed, 70 (55.12%) serine proteases and 42 (50.0%) protease inhibitors (Table S1). An analysis revealed that these orthologous genes are involved in 409 distinct pathways or processes, including a key subset (~15%) involving E3 ubiquitin ligase ubiquitinate target proteins (R-HSA-8866654), protein ubiquitination (R-HSA-8852135), post-translational protein modification (R-HSA-597592) and protein metabolism (R-HSA-392499) (Table S3).

### 2.6. Diverse Transcription and Stage- and Sex-Specific Patterns

**Figure 3:**
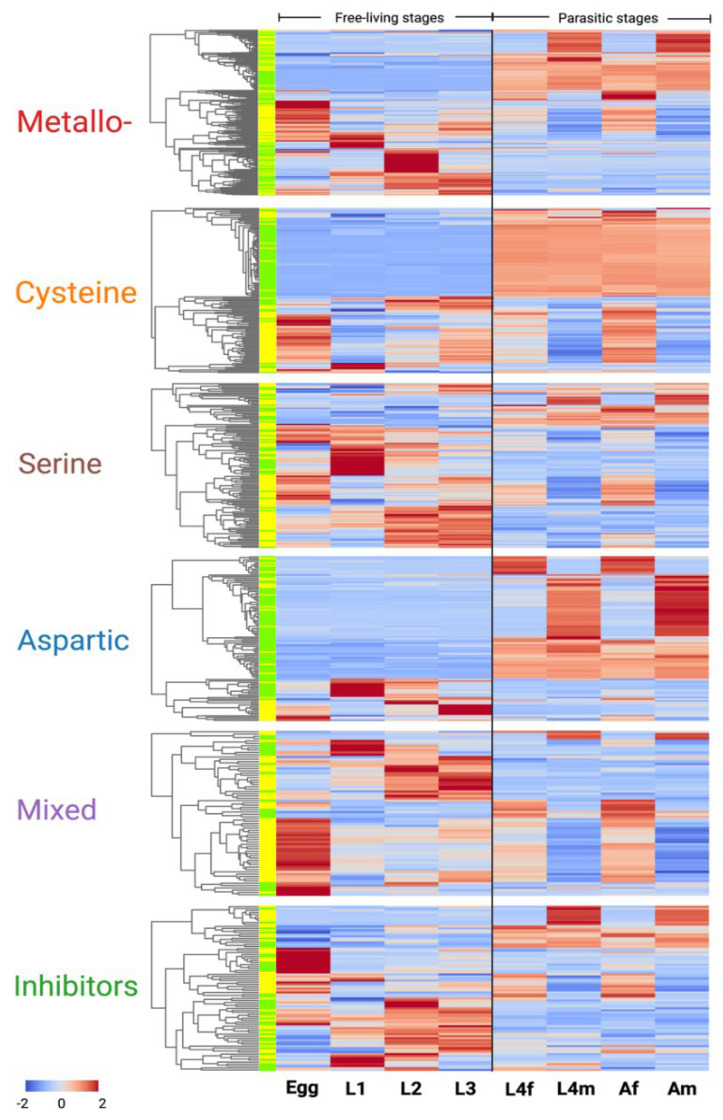
Transcription profiles of protease and protease inhibitor genes in different developmental stages of *Haemonchus contortus*. Free-living stages: egg; first-, second- and third-stage larvae (i.e., L1, L2 and L3; and free-living stages). Parasitic stages: female and male fourth-stage larvae (L4f and L4m); and female and male adults (Af and Am). Left: clustering of genes based on transcription profiles, with green indicating genes encoding excretory/secretory (ES) proteins and yellow denoting other (i.e., non-ES) proteins. Right: transcription profiles of individual genes. The blue to red scale indicates normalised fragments per kilobase per million (FPKM) values.

The transcriptional profiles of genes encoding proteases and protease inhibitors of *H. contortus* were investigated across all key developmental stages/sexes, including egg, L1, L2 and L3, L4f, L4m, Af and Am. Metallo-protease genes were represented in four main clusters, based on markedly distinct transcription patterns. Specifically, 39 genes were highly transcribed in parasitic stages (i.e., L4f, L4m, Af and Am), 28 genes displayed male-specific transcription (in L4m and Am), 11 genes exhibited relatively high transcription levels in the egg stage and 28 genes had a high transcription level in the L2 stage. Notably, most genes transcribed in parasitic stages (L4f, L4m, Af and Am) encoded excretory/secretory (ES) proteins, whereas those with high transcription in free-living stages (egg, L1, L2 and L3) encoded proteins that are not ES proteins (Figure 3).

Almost half of all cysteine protease genes (*n* = 61) exhibited high transcription in the parasitic stages (i.e., L4f, L4m, Af and Am), 90% of which encode ES proteins. Two gene clusters were identified, with five genes exhibiting transcriptional activity in the egg, and five genes with high transcription in the L1 stage (Figure 3).

In contrast to metallo-protease and cysteine protease genes, there were no serine protease gene clusters exhibiting high transcription in the parasitic stages of *H. contortus*. However, three distinct clusters of six genes each exhibiting high, male-specific transcription in L4m and Am, 17 genes exhibiting high transcription in L1, and 31 genes displaying high transcription in the L2 and L3 stages were identified. Most (76.5%) of these 17 protease genes with high transcription levels in the L1 stage encoded ES proteins, whereas most such genes with high expression in the L2 and L3 stages encoded non-ES proteins (Figure 3).

The transcription profiles of aspartic protease genes were diverse among the different developmental stages/sexes of *H. contortus*. Twelve and 38 genes exhibited high transcription in the parasitic female (i.e., L4f and Af) and male (i.e., L4m and Am) stages. Additionally, 17 genes exhibited high transcription in both sexes of the parasitic stages (i.e., L4f, L4m, Af and Am), nine genes in L1, and seven genes in the L3 stage (Figure 3).

For the ‘mixed’ protease genes, clusters were not as clearly defined as for other protease genes. However, 27 and 7 genes were highly transcribed in the egg and L1 stages, respectively. The former group represented predominantly ES proteins, whereas the latter represented mainly non-ES proteins. Intriguingly, most (83.3%) mixed protease genes were transcribed in free-living stages, which contrasts the findings for other protease gene groups. These findings suggest that mixed protease genes might play key roles, particularly in free-living stages of *H. contortus* (Figure 3).

Protease inhibitor genes also exhibited conspicuous transcription profile differences among developmental stages, with three distinctive gene clusters observed. Specifically, 10 inhibitor genes displayed high transcription in male (L4m and Am) parasitic stages, most of which coded for ES proteins; 13 genes were highly transcribed in the egg, half of which code for ES proteins; and five genes were highly transcribed in the L1 stage (Figure 3).

## 3. Discussion

Proteases have consistently been a focus in biological research due to their potential as vaccine and therapeutic targets [62,63,64], particularly in the context of controlling parasitic infections or diseases [18,65,66]. Although numerous studies have employed molecular techniques to identify and investigate particular proteases of *H. contortus*, such as *Hc*-CBL (HC58), *Hc*-CPL-1 and *Hc*-CBP-1 [59,67,68], there had been no comprehensive, systematic analysis of these proteins. Recent advances in genomics, transcriptomics, proteomics [43,44,45,46,47,48] and informatics, including artificial intelligence-based structural modelling [25,69,70,71], have created new avenues for the characterisation of proteases encoded within the *H. contortus* genome. Thus, using the curated data available in MEROPS database, we employed a combination of sequence- and structure-based methods to identify 790 genes encoding 860 proteases or protease inhibitors (including distinct isoforms) in the *H. contortus* genome. The proteases were shown to belong to 83 distinct gene families and displayed considerable variation in biophysical properties and in the presence of signal peptides. These findings offer valuable insights into the diversity of *H. contortus* proteases and their inhibitors, establishing a foundation for functional investigations of these molecules and for discovering potential novel drug or vaccine targets.

### 3.1. Combining Structure- and Sequence-Based Methods Enhances the Identification of Proteases and Their Inhibitors

As distinct from conventional methods that rely solely on sequence-based identification [9,24], the present study integrates sequence homology and structural similarity to accurately identify proteases and their inhibitors in *H. contortus*. Using this approach, we were able to detect 805 homologous proteins based on sequence similarity alone, and 401 proteins using structural similarity. Using both techniques, 860 proteins were identified, markedly enhancing the overall number (cf. Section 2.1).

Of the 860 proteins identified, 346 exhibited homologous relationships with proteins in the MEROPS database at both the sequence and structure levels [8]. For instance, protein HCON_00016690-00002 had a high sequence similarity (E-value < 10^−109^) with the metallo-protease MER0001185 in this database, and is an orthologue of *C. elegans* WBGene00009865 [72,73]. Additionally, it exhibits a structural similarity of TM = 0.96 with human endoplasmic reticulum aminopeptidase 2YD0 in the PDB database [74]. This information provides confidence about the identity of this molecule.

We discovered 55 proteins based on structural similarity alone. These proteins lacked homologues at the sequence level, but matched proteins with known structures [75,76,77] in MEROPS. For instance, protein HCON_00150390-00001 did not share Diamond/BLASTP homology to a protein in the MEROPS sequence search, but its predicted three-dimensional structure matched protease inhibitor BIRC-7 (TM = 0.93), clearly indicating that it is a protease inhibitor [78]. This result shows that a structure-based method can sometimes overcome limitations inherent in a sequence-based identification method [79,80,81].

The identification of 459 proteins exclusively using the sequence-based method highlights a limitation of structure-based identification alone [82,83,84], which may relate to the incompleteness of protein structure databases [85,86,87,88]. On the one hand, not all proteases in the MEROPS database are linked to structural information. On the other hand, only 66% of structures of *H. contortus* proteins predicted by AlphaFold2 were of an acceptable quality (pLDDT of > 70). Thus, we propose that a combined sequence- and structure-based approach achieves superior protein identification. Nonetheless, it is crucial that databases and informatic approaches are continually updated to allow high-quality identification and subsequent in silico classification and functional annotation.

### 3.2. Key Protease Gene Families Associate with Key Biological Functions in Parasite Invasion and Feeding as Well as Host–Parasite Interactions, Immunomodulation and Parasitism

The distribution of *H. contortus* proteases (according to families) was similar to that observed in the MEROPS database for *C. elegans* and other model metazoans [8], which provides evidence in support of the hypothesis that the ‘protease system’ has remained relatively constant throughout evolution (cf. [24,89,90]). However, we also noted some differences/discrepancies, particularly with respect to particular gene families that possess many members (e.g., A01, C01 and M12). This may relate to gene family expansions during the course of evolution [91,92,93], which would/could have facilitated adaptation to the unique parasitic lifestyle of *H. contortus*. Therefore, members of these families may be potential intervention targets, warranting future investigation.

The aspartic protease family A01 of *H. contortus* consists of a remarkably large number of proteins—a total of 98 (see Section 2.2). Such proteases possess the ability to cleave peptide bonds using two aspartic acid residues located in their active sites [94]. Previous studies of model species (e.g., *C. elegans* and *D. melanogaster*) revealed that this family includes pepsin, renin and cathepsin D [95,96,97]. In parasites, aspartic proteases of family A01 can play critical roles in the processing of surface proteins required for host cell invasion and/or immune evasion [98,99]. Additionally, these proteases contribute to the degradation of host proteins, which helps the parasites obtain nutrients from their host environment [100]. In helminths, these proteases are vital in breaking down host proteins, such as antibodies, facilitating a parasite’s invasion and establishment in the host animal [101,102,103]. This information indicates that these A01 proteases are critical for the *H. contortus* parasitic life. In addition, the transcriptional data also support this observation, where many *H. contortus* A01 protease genes encode ES proteins and are highly transcribed/expressed in the L4 and adult stages (i.e., Af and Am; Figure 2), indicating a role in host invasion and survival within the host.

Of the cysteine proteases identified in *H. contortus*, family C01 contains the largest number of proteins—a total of 84 (see Section 2.2). C01 proteases are known for their diverse functions in protein-turnover, antigen processing, apoptosis, inflammation and pathogenesis [104,105,106]. One of the significant characteristics of C01 proteases in parasites is their high degree of diversity [107,108]. For example, blood flukes, such as *Schistosoma mansoni*, have large numbers of C01 proteases that differ markedly in their sequence, structure and substrate specificity (e.g., [20,109]). This diversity allows parasites to efficiently degrade and utilise a wide range of host proteins and tissues [110,111]. Interestingly, almost all C01 family proteases identified in *H. contortus* are ES proteins, whose genes are highly transcribed in the parasitic stage (Figure 2). This finding indicates that *H. contortus* secretes C01 family proteases, which could act as virulence factors by degrading host proteins, disrupting host tissues and modulating host immune responses in the host animal (cf. [19,67]).

Metallo-protease family M12 contains the largest number of proteases in *H. contortus*, with a total of 130 members (cf. Section 2.2). This family of proteases is characterised by the presence of a conserved zinc-binding motif in their catalytic domains and plays crucial roles in various biological processes, including tissue remodelling and/or the regulation of signalling pathways [112,113]. In helminths, a variety of M12 proteases have been inferred or shown to be involved in the degradation of host tissues and in immune evasion [114]. In *H. contortus*, genes encoding M12 proteases were shown to be highly transcribed in all developmental stages, indicating important roles throughout the life cycle of this parasite (Figure 2).

Interestingly, some proteases belonging to the families A01, C01 and M12 are known to play a critical role in the blood-feeding pathways of parasites, as they participate in the proteolytic cascade of haemoglobin degradation [115,116]. It has been postulated that proteases of the family A01 represent the initial step in host haemoglobin degradation [18], such that host haemoglobin is first targeted by aspartic proteases and then degraded into smaller peptides by cysteine proteases (including those of family C01) [117]. This process is followed by further degradation into smaller peptides by metallo-proteases, including those of family M12 [12]. As haemoglobin degradation serves as the primary source of nutrition for *H. contortus* and is vital for its survival in the parasitic phase within the host, the expanded number of M12 protease genes might be explained by the parasite’s need for a diverse array of proteases to maintain a ‘stability’ in blood-feeding pathway and to suppress immune attack by the host animal (cf. [18,115]). The disruption or interruption of the functions of such proteases markedly affects a haematophagous parasite’s ability to acquire critical nutrients from blood, which is why previous efforts have targeted selected proteases as vaccine molecules or candidates (e.g., [118,119,120,121,122,123,124]). For example, the metallo-protease (family M01) represented by HCON_00156280 [125] is a membrane-bound glycoprotein H11 and a known vaccine molecule. When assessed as a vaccine, H11 reduced total worm numbers of adult *H. contortus* in vaccinated sheep by 70–80%, and associated egg numbers in faeces by >90% [119,125,126,127,128]. H11 is also a component of Barbervax^®^—the first licensed, native anti-*H. contortus* vaccine, launched in Australia (https://barbervax.com/; refs. [125,129,130]). Thus, there is clear merit in pursuing further research on metallo-proteases of *H. contortus* to identify well-defined immunogens, recognising that, sometimes, immunogenicity might be linked to post-translational (e.g., carbohydrate) moieties [131].

### 3.3. The Biological Relevance and Meaning of Diverse Transcriptional Profiles for Protease and Protease Inhibitor Genes—Significance and Implications

The genes that encode proteases and protease inhibitors in *H. contortus* displayed varied transcription patterns among developmental stages (Section 2.6). In free-living (i.e., egg, L1, L2 and L3) stages, transcription levels of genes encoding, for example, M12, C12, C13, A22, and T01 proteases, were high, particularly in the egg stage, suggesting key roles in embryonic development (cf. [132]). In the L1, L2 and L3 stages, genes encoding, for example, M12 proteases, have been proposed to be linked to larval growth and development and/or moulting [133,134].

In parasitic stages (i.e., L4f, L4m, Af and Am), more than 100 genes encoding metallo-, cysteine, serine and aspartic proteases (Section 2.6) were highly transcribed, contrasting the distinctly different profiles of orthologues in the free-living nematode *C. elegans* (see [135,136]), and are thought to be linked to parasite invasion, establishment and/or survival. These genes encoded predominantly metallo-proteases (particularly family M12), cysteine proteases (mostly family C01) and aspartic proteases (largely family A01) (Table S1), many of which are likely involved in immunomodulation [59,60] or in the blood-feeding pathway, which has been the subject of significant previous investigation in *H. contortus* (Section 3.2; [67,137,138]). Moreover, metallo-, cysteine and aspartic proteases, such as MEP1, HMCP-1, HMCP-4, HMCP-6, pep1 and H11, have been recognised as key participants in the haemoglobin degradation process [56,115,120,121,139,140,141], which makes many of them possible vaccine and drug target candidates.

In female parasitic stages (L4f and Af) of *H. contortus*, highly transcribed genes encoding aspartic proteases of family A01 and cysteine proteases of family C19 (Table S1), such as the ubiquitin-specific proteases (USPs), highly likely play a key role in regulating the ubiquitin-proteasome system (UPS), which are critically involved in protein turnover [32,142]. These proteases appear to be implicated in processes linked to sexual development, maturation and/or reproduction [143], which is why they may represent targets for the disruption of these genes/proteases (cf. [144]). In male parasitic stages (L4m and Am) of *H. contortus*, highly transcribed protease genes encoded predominantly aspartic proteases (family A01), serine proteases (families S09 and S12) and metallo-proteases (family M18) (Section 2.6). Given their apparent male-specificity, we propose that these molecules play one or more critical role(s) in male reproductive development or sperm production in this worm, supported by published evidence of the involvement of similar proteases in sperm maturation and function in various other eukaryotic organisms [145,146,147]. This information on sex-enriched transcription/expression of particular proteases provides a clear stimulus to explore the roles of these proteins in the reproductive processes in *H. contortus*, and also to assess their potential as intervention targets.

## 4. Materials and Methods

### 4.1. Identification, Classification and Annotation of Proteases and Protease Inhibitors of H. contortus

The latest genome and proteome of *H. contortus* (ISE strain; ref. [48]) were downloaded from WormBase ParaSite (version WBPS17; accession: PRJEB506; https://parasite.wormbase.org/; accessed on 15 February 2023; ref. [148]). Relevant information (including identifiers, classes and families) of proteases (including aspartic, cysteine, glutamic, “mixed”, metallo-, asparagine, serine and threonine representatives) was retrieved from the MEROPS database (https://www.ebi.ac.uk/merops/; accessed on 15 February 2023; ref. [8]) to assist the identification, classification and annotation of proteases and their inhibitors in *H. contortus*. Both sequence- and structure-based search methods were utilised for this purpose.

For the sequence-based strategy, the protease and protease inhibitor protein sequences from the MEROPS database were used as queries to identify homologues in *H. contortus* using the program BLAST v.2.12.0 [149], employing a sequence identity threshold (E-value) for BLAST of <10^−5^. InterProScan v.5.55-88.0 [150] was used to detect relevant Pfam [151] and PANTHER [152] domains using default settings. For the structure-based strategy, known protease and protease inhibitor structures—collected from, and documented in, MEROPS, and downloaded from PDB [153]—were used as queries to search against our own, in-house *H. contortus* protein structure database to establish structural homology using US-align v.20220904 [154]. US-align’s threshold (TM-score) was set at >0.5. The structures of mature proteases were predicted using AlphaFold2 [70]. Individual proteins and their gene models were manually inspected and curated [49]. Functional annotation was achieved employing a recently established pipeline, with all proteins being assigned gene ontology (GO) terms [51]. Excretory/secretory (ES) proteins were inferred according to previous studies [43,51].

### 4.2. Prediction of Biophysical Properties and Sequence Features

The physicochemical properties of identified proteases and protease inhibitors, including molecular weight (in kDa), isoelectric point (pI), charge (at pH 7.0), grand average of hydropathy (GRAVY) and instability index (II), were estimated using EXPASy (https://www.expasy.org/; refs. [155,156]) and Biopython (https://biopython.org/) [157]. Signal peptides were predicted using SignalP v.6.0 [158]. Conserved domains in proteins were investigated using InterProScan v.5.55-88.0 [150]. Protease-encoding genes identified were mapped to the *H. contortus* chromosome (ISE strain; accession: PRJEB506; ref. [48]) and displayed using TBtools v.1.09876 [159].

### 4.3. Detection of Orthologues and Pathway Analysis

OrthoFinder v.2.5.4 [160] was used to find orthologues of *H. contortus* proteases and protease inhibitors in *C. elegans*. Based on the information from *C. elegans* orthologues, we inferred pathway information linked to orthologues using the Reactome Pathway Knowledgebase (https://reactome.org/; ref. [161]).

### 4.4. Gene Transcription and Protein Expression

Transcription profiles for key developmental stages/sexes—i.e., egg, first-stage larva (L1), second-stage larva (L2), third-stage larva (L3), female (L4f) and male (L4m) fourth-stage larva (L4) and female (Af) and male (Am) adult—were available from previous studies [40,162]. RNA-seq data were processed and analysed using trim_galore v.0.6.5 (https://zenodo.org/badge/latestdoi/62039322, accessed on 15 February 2023), SAMtools v.1.13 [163], MultiQC v.1.10 [164], HISAT2 v.2.2.0 [165] and StringTie v.2.0.5 [165], and the transcript abundances of identified protease genes, expressed as fragments per kilobase per million (FPKM) [32], were calculated. Custom Python scripts were used to establish normalised transcription levels of individual genes.

The expression levels of proteases and protease inhibitors in *H. contortus* across different developmental stages were available from a previous study [43,44]. Proteome discoverer software [166] was used to search mass spectrometric data for the egg, L3, L4 and adult stages. Peptides were identified using a false discovery rate (FDR) cut-off of <1%, and peptide intensities were calculated with Spectronaut software. At least two matching peptides were required for expression to be recorded, and protein expression levels were expressed as ‘intensities’. Protein phosphorylation was explored with reference to published data sets [45,49].

## 5. Conclusions

Genetic and genomic variability both within and among species in the Tree of Life present major challenges for the accurate identification, characterisation, classification and functional annotation of molecules in different taxonomic groups of animals, particularly invertebrates. In order to overcome such challenges associated with proteases and protease inhibitors, we established here an effective bioinformatic workflow that utilises a combination of both sequence- and structure-based in silico methods. We employed curated genomic, transcriptomic and proteomic data sets for the well-characterised model parasitic nematode, *H. contortus*, to effectively identify, classify and functionally annotate proteases and protease inhibitors of this species on a genome-wide scale. Although established and critically assessed here for *H. contortus*, this workflow should now find broad applicability to any eukaryotic pathogen, enabling both fundamental biodiscovery and the search for new intervention targets in socio-economically important pathogens.

## Figures and Tables

**Table 1:** Distribution of protease and protease inhibitor genes, proteins (isoforms) and protein families in *Haemonchus contortus*.

Class/Group	Number of Genes	Number of Proteins (Including Isoforms)	Number of Protein Families
Aspartic proteases	115	121	4
Cysteine proteases	133	145	20
Metallo-proteases	259	280	21
Serine proteases	127	142	12
‘Mixed’ proteases	72	81	12
Protease inhibitors	84	91	14
Totals:	790	860	83

## Data Availability

The predicted protein structure data are accessible via https://figshare.com/s/f8cff0e89caa31ff98e9 (accessed on 30 May 2023).

## Supplementary Materials

The following supporting information can be downloaded at: https://www.mdpi.com/article/10.3390/ijms241512320/s1.
